# Evaluating the Quality of Research into a Single Prognostic Biomarker: A Systematic Review and Meta-analysis of 83 Studies of C-Reactive Protein in Stable Coronary Artery Disease

**DOI:** 10.1371/journal.pmed.1000286

**Published:** 2010-06-01

**Authors:** Harry Hemingway, Peter Philipson, Ruoling Chen, Natalie K. Fitzpatrick, Jacqueline Damant, Martin Shipley, Keith R. Abrams, Santiago Moreno, Kate S. L. McAllister, Stephen Palmer, Juan Carlos Kaski, Adam D. Timmis, Aroon D. Hingorani

**Affiliations:** 1 Department of Epidemiology and Public Health, University College London, United Kingdom; 2 Department of Health Sciences, University of Leicester, United Kingdom; 3 Centre for Health Economics, University of York, United Kingdom; 4 St George's University of London, United Kingdom; 5 Barts and the London Medical School, United Kingdom; The George Institute, Australia

## Abstract

**Background:**

Systematic evaluations of the quality of research on a single prognostic biomarker are rare. We sought to evaluate the quality of prognostic research evidence for the association of C-reactive protein (CRP) with fatal and nonfatal events among patients with stable coronary disease.

**Methods and Findings:**

We searched MEDLINE (1966 to 2009) and EMBASE (1980 to 2009) and selected prospective studies of patients with stable coronary disease, reporting a relative risk for the association of CRP with death and nonfatal cardiovascular events. We included 83 studies, reporting 61,684 patients and 6,485 outcome events. No study reported a prespecified statistical analysis protocol; only two studies reported the time elapsed (in months or years) between initial presentation of symptomatic coronary disease and inclusion in the study. Studies reported a median of seven items (of 17) from the REMARK reporting guidelines, with no evidence of change over time.

The pooled relative risk for the top versus bottom third of CRP distribution was 1.97 (95% confidence interval [CI] 1.78–2.17), with substantial heterogeneity (I^2^ = 79.5). Only 13 studies adjusted for conventional risk factors (age, sex, smoking, obesity, diabetes, and low-density lipoprotein [LDL] cholesterol) and these had a relative risk of 1.65 (95% CI 1.39–1.96), I^2^ = 33.7. Studies reported ten different ways of comparing CRP values, with weaker relative risks for those based on continuous measures. Adjusting for publication bias (for which there was strong evidence, Egger's *p*<0.001) using a validated method reduced the relative risk to 1.19 (95% CI 1.13–1.25). Only two studies reported a measure of discrimination (c-statistic). In 20 studies the detection rate for subsequent events could be calculated and was 31% for a 10% false positive rate, and the calculated pooled c-statistic was 0.61 (0.57–0.66).

**Conclusion:**

Multiple types of reporting bias, and publication bias, make the magnitude of any independent association between CRP and prognosis among patients with stable coronary disease sufficiently uncertain that no clinical practice recommendations can be made. Publication of prespecified statistical analytic protocols and prospective registration of studies, among other measures, might help improve the quality of prognostic biomarker research.

*Please see later in the article for the Editors' Summary*

## Abstract 2

In a systematic review and meta-analysis of 83 prognostic studies of C-reactive protein in coronary disease, Hemingway and colleagues find substantial biases, preventing them from drawing clear conclusions relating to the use of this marker in clinical practice.

## Editors' Summary

**Background:**

Coronary artery disease is the leading cause of death among adults in developed countries. With age, fatty deposits called atherosclerotic plaques coat the walls of the arteries, the vessels that carry blood to the body's organs. Because they narrow the arteries, atherosclerotic plaques restrict blood flow. If plaques form in the arteries that feed the heart, the result is coronary artery disease, the symptoms of which include shortness of breath and chest pains (angina). If these symptoms only occur during exertion, the condition is called stable coronary artery disease. Coronary artery disease can cause potentially fatal heart attacks (myocardial infarctions). A heart attack occurs when a plaque ruptures and a blood clot completely blocks the artery, thereby killing part of the heart. Smoking, high blood pressure, high blood levels of cholesterol (a type of fat), diabetes, and being overweight are risk factors for coronary artery disease. Treatments for the condition include lifestyle changes and medications that lower blood pressure and blood cholesterol. Narrowed arteries can also be widened using a device called a stent or surgically bypassed.

**Why Was This Study Done?:**

Clinicians can predict whether a patient with coronary artery disease is likely to have a heart attack by considering their risk factors. They then use this “prognosis” to help them manage the patient. To provide further help for clinicians, researchers are trying to identify prognostic biomarkers (molecules whose blood levels indicate how a disease might develop) for coronary artery disease. However, before a biomarker can be used clinically, it must be properly validated and there are concerns that there is insufficient high quality evidence to validate many biomarkers. In this systematic review and meta-analysis, the researchers ask whether the evidence for an association between blood levels of C-reactive protein (CRP, an inflammatory protein) and subsequent fatal and nonfatal events affecting the heart and circulation (cardiovascular events) among patients with stable coronary artery disease supports the routine measurement of CRP as recommended in clinical practice guidelines. A systematic review uses predefined criteria to identify all the research on a given topic; a meta-analysis is a statistical method for combining the results of several studies.

**What Did the Researchers Do and Find?:**

The researchers identified 83 studies that investigated the association between CRP levels measured in people with coronary artery disease and subsequent cardiovascular events. Their examination of these studies revealed numerous reporting and publication short-comings. For example, none of the studies reported a prespecified statistical analysis protocol, yet analyses should be prespecified to avoid the choice of analytical method biasing the study's results. Furthermore, on average, the studies only reported seven of the 17 recommended items in the REMARK reporting guidelines, which were designed to improve the reporting quality of tumor biomarker prognostic studies. The meta-analysis revealed that patients with a CRP level in the top third of the distribution were nearly twice as likely to have a cardiovascular event as patients with a CRP in the bottom third of the distribution (a relative risk of 1.97). However, the outcomes varied considerably between studies (heterogeneity) and there was strong evidence for publication bias—most published studies were small and smaller studies were more likely to report higher relative risks. Adjustment for publication bias reduced the relative risk associated with high CRP levels to 1.19. Finally, nearly all the studies failed to calculate whether CRP measurements discriminated between patients likely and unlikely to have a subsequent cardiovascular event.

**What Do These Findings Mean?:**

These findings suggest that, because of multiple types of reporting and publication bias, the size of the association between CRP levels and prognosis among patients with stable coronary artery disease is extremely uncertain. They also suggest that CRP measurements are unlikely to add anything to the prognostic discrimination achieved by considering blood pressure and other standard clinical factors among this patient group. Thus, the researchers suggest, the recommendation that CRP measurements should be used in the management of patients with stable coronary artery disease ought to be removed from clinical practice guidelines. More generally, these findings increase concerns about the quality of research into prognostic biomarkers and highlight areas that need to be changed, the most fundamental of which is the need to preregister studies on prognostic biomarkers and their analytic protocols.

**Additional Information:**

Please access these Web sites via the online version of this summary at http://dx.doi.org/10.1371/journal.pmed.1000286.

- The MedlinePlus Encyclopedia has pages on coronary artery disease and C-reactive protein (in English and Spanish)
- MedlinePlus provides links to other sources of information on heart disease
- The American Heart Association provides information for patients and caregivers on all aspects of cardiovascular disease, including information on the role of C-reactive protein in heart disease
- Information is available from the British Heart Foundation on heart disease and keeping the heart healthy
- Wikipedia has pages on biomarkers and on C-reactive protein (note that Wikipedia is a free online encyclopedia that anyone can edit; available in several languages)
- The EQUATOR network is a resource center for good reporting of health research studies

## Introduction

### What Is the Problem?

Robust research evidence on the prognostic value of circulating biomarkers is important for translational medicine and clinical decision making, but there are concerns about the quality of such evidence [1], largely based on studies in the field of cancer. Systematic reviews and meta-analyses across multiple cancer biomarkers [2]–[4] have found biases arising from selection of studies for publication, or selection of findings for inclusion within published studies. There have been few evaluations of the quality of evidence focussing on a single biomarker [5]. It is not known the extent to which such biases threaten validity of putative prognostic biomarkers among people with cardiovascular disease, because of a lack of large scale evaluations. Indeed in healthy population studies of cardiovascular disease onset [6], reliable associations largely free of such biases with a range of biomarkers have been demonstrated. We studied C-reactive protein (CRP) in the prognosis of stable coronary artery disease because it is the most widely investigated (>100 studies) novel prognostic biomarker in such patients [7], and therefore the research might be expected to have reached reliable conclusions. Furthermore, there is clinical uncertainty as to whether to measure CRP, with US [8] and European [9] clinical practice guidelines recommending measurement, but clinical practice varying widely [10].

### Objectives

In evaluating the quality of published evidence on CRP in the prognosis of patients with stable coronary disease we carried out a systematic review, meta-analysis, and meta-regression [11],[12] with five specific objectives: (i) To determine the quality of study reporting based on a systematic review. In the absence of agreed criteria for measuring the quality of reporting we extended previous efforts [3], and operationalised reporting guidelines for tumour markers [12] into 17 items. A particular concern of ours [1], notably absent from reporting guidelines, was whether studies reported a reference to a study protocol or prespecified statistical analytic protocol; (ii) To determine the extent to which any association of CRP on prognosis was independent of established prognostic factors. Unlike many cancers, cardiovascular disease has numerous established markers of prognosis that are also associated with aetiology, and CRP is a good example of a prognostic biomarker that is highly correlated with these (smoking, diabetes, obesity, lipids, and other markers of inflammation, such as fibrinogen) [13],[14]. The impact of biases in incomplete adjustment for established risk factors has seldom been assessed in large meta-analyses of prognostic biomarkers; (iii) To determine the presence and magnitude of bias arising from small studies. While previous meta-analyses have highlighted that publication bias exists, here we use recently validated methods to assess the potential magnitude of such biases [15]; (iv) To explore sources of heterogeneity, particularly to assess whether aspects of study design or reporting influenced the summary estimate of effect; (v) To determine the extent to which papers report the ability of CRP to discriminate patients who do and do not experience subsequent events. Reporting such data has recently been recommended [16], but it is not known how commonly it is reported.

## Methods

### Search for Eligible Papers and Inclusion Criteria

We included any prospective observational study (observational cohort studies, prospective nested case control studies, observational data drawn from randomised controlled trials) that reported risk of subsequent events among patients with stable coronary disease in relation to measured CRP values. Eligible studies had to include patients with stable coronary disease, defined as clinically diagnosed angina pectoris or angiographic disease, or a history of previous acute coronary syndrome at least 2 wk prior to CRP measurement. We excluded studies where CRP was measured only during an admission with an acute coronary syndrome, or only after a coronary procedure, but before discharge. Eligible outcome events were defined as coronary (coronary death, sudden cardiac death, acute nonfatal myocardial infarction, primary percutaneous coronary intervention, unplanned emergency admissions with unstable angina), cardiovascular (where coronary events were reported in combination with heart failure, stroke, or peripheral arterial disease), and all cause mortality alone. We did not exclude any studies on the basis of methodological standards, sample size, duration of follow-up, publication year, or language of publication. We searched MEDLINE (PubMed) between 1966 and 25 November 2009 and EMBASE between 1980 and 17 December 2009 databases using a strategy developed with an expert librarian based on terms for coronary disease (from the Cochrane Library of systematic reviews and protocols), prognostic studies [17], and CRP. Three reviewers (NKF, JD, KM) reviewed article titles and abstracts for eligibility and obtained full text articles where eligibility was definite or unclear (see Figure S1).

### Data Extraction for Systematic Review

The two reviewers independently abstracted data from eligible articles (*n = *116) using a predefined coding protocol. Non-English articles were translated (*n = *4). Individual item disagreement between the two reviewers was resolved by consensus or, rarely, adjudication by a third reviewer (HH). We extracted information on year of publication, year of study start, number of patients at baseline that were included in the analysis, their mean age and percent women, the baseline coronary morbidity (proportion with stable angina, angiographic disease, or previous myocardial infarction), average levels of biomarker at baseline (either mean [SD] or median [interquartile (IQR) range]) in the whole sample and separately among those who did and did not subsequently experience an outcome event, and type of high sensitivity CRP assay, follow-up duration, the number and type (coronary, cardiovascular, and all cause mortality) of outcome events (from which the crude annual risk was calculated).

### Data Extraction for Quality of Study Reporting

We developed closed-ended questions to operationalise prognostic biomarker reporting guidelines [12] and extracted details on 17 items (see Table S1) relating to prespecified research question, population at start and end of follow up, biomarker measurement, outcome assessment, confounder measurement, and analytic choices.

### Data Extraction for Relative Risks

We extracted the reported relative risk, odds ratio or hazard ratio, and 95% confidence intervals (CIs) from each study. Where one study had multiple eligible articles or one article reported multiple relative risks we extracted the relative risks for the most specific coronary outcome event (according to the hierarchy coronary, cardiovascular, all cause mortality) with the largest number of adjustment variables. Where available we extracted separate relative risk estimates with different degrees of confounder adjustment for the following prespecified conventional risk factors (age, sex, smoking status, obesity, diabetes, and one or more lipid variables [from total cholesterol, LDL cholesterol, HDL cholesterol, trigylcerides], and inflammatory markers [fibrinogen, IL-6, white cell count]).

### Statistical Analysis

We converted the reported relative risk estimates onto a standard scale of effect, comparing the highest third with the lowest third of the CRP distribution, in essence giving an estimate per 2.18 SD units of CRP where 2.18 is the difference in the means of the top and bottom third of the standard normal distribution [18]. The reported comparisons included continuous measures (per SD, tertile, quartile, unit [mg/l] on original or log 10 scale), equal size groups (top versus bottom with group size 50%, 33%, or 25% for 2, 3, and 4 groups, respectively), unequal size groups (top versus bottom; 2 groups, 3 groups defined by cut-points), as well as measures on both log-transformed and untransformed CRP scales. The scaling methods assume that CRP is log normally distributed and that the association with disease risk is log-linear; both these assumptions have empirical support in healthy population studies of CRP [19],[20]. For two equal groups the difference in means is 1.59 SD units and we used a scaling factor of 1.37 (2.18/1.59). For four and five groups we used a scaling factor of 2.18/2.54 and 2.18/2.80, respectively, i.e., the difference in means between the top and bottom tertile in each case under the assumption of log normality for CRP. Unequal groups required study-specific scaling factors, which were calculated as 2.18/*x* where *x* is the difference in means between the unequal groups. The differences were found by simulating one million observations from the distribution used to report the comparison (i.e., normal or log normal). For normally transformed CRP, relative risks reported per standard deviation used a scaling factor of 2.18 and relative risks reported per unit were converted first to a SD change, using the study specific SD and thence to tertiles. For untransformed CRP, relative risks reported per standard deviation were scaled using the study-specific difference in means between the upper and lower tertiles and the SD, and those reported per mg/l were scaled using the difference in means alone.

### Statistical Methods for Meta-analysis and Meta-regression

For each study, the relative risk estimate and its corresponding standard error were transformed to their natural logarithms to stabilise the variance and to normalise the distributions. Summary relative risk estimates and their 95% CIs were estimated from a random effects model that considers both within- and between-study variation [21]. Statistical heterogeneity among studies was evaluated using the I^2^ statistic [22].

Small study (including publication) bias was assessed with contour-enhanced funnel plots (i.e., a plot of study relative risk estimate against precision, with contours representing varying levels of statistical significance), by Begg's adjusted rank correlation test, and by Egger's regression asymmetry test [23],[24]. We used previously investigated methods to adjust the meta-analyses for the potential impact of publication bias (see Table S3) [25]. These included; (i) “trim and fill,” an iterative nonparametric method using a rank-based data augmentation technique to account for asymmetry in the funnel plot. Both the “trimming” of asymmetric studies, for which there are no counterparts, and the revised pooled estimate after “filling” (or imputing) these counterparts can be based on either a fixed or random effects meta-analysis model. Models considered here use either fixed or random effects models for both components, or fixed effect model to “trim” and random effects to “fill.” (ii) Weighted regression-based methods, which are extensions of Egger's regression asymmetry test [24],[25] and regress the outcome against a measure of study precision (standard error, variance, or sample size), weighted by either the reciprocal of either the total variance or the variance of the proportion of the number of events in a study, in order to predict the effect size in a (hypothetically) infinitely large study as a pooled estimate adjusted for publication bias. These meta-regression models can either be fixed effect or random effects models, or can allow for between-study variability via a dispersion parameter. (iii) Conditional regression-based methods, in which a test for small study bias is performed first, and then if appropriate, regression-based methods (as previously described above) are used to adjust the observed effect size [25]. A quadratic version of the original Egger regression test (using the variance rather than the standard error) and including allowing for between-study variability via a dispersion parameter has been shown in both simulation [25] and empirical [15] studies to out-perform other approaches.

To explore other potential sources of study heterogeneity, we employed a random effects meta-regression model that included study level continuous or categorical covariates. Assumptions of normality, independence, and homogeneity of residuals were verified via diagnostic plots.

### Discrimination

We calculated the detection rate at different false positive rates by constructing the log-normal distributions of CRP separately for those with and without outcome events using previously reported methods [26],[27]. Calculating the detection rate for false positive rates from 0 to 100 then yields a receiver operating characteristic (ROC) curve for the outcome group, from which c-statistics can be calculated using the trapezium rule. Confidence intervals for the ROC curves and detection rate at the 10% false positive rate were obtained using large sample properties of binormal ROC curves [28] and pooled estimates of both the c-statistic and detection rate were subsequently obtained by random effects meta-analysis of the study-specific c-statistics and detection rates. All analyses were conducted using Stata, version 10.0 (StataCorp). All statistical tests were 2-sided.

## Results

### Systematic Review

We identified 1,566 articles of which 83 studies fulfilled our inclusion criteria (Figure S1) and are summarised in the systematic review (Table S1). There were a total of 61,684 patients and 6,485 outcome events in these studies (median per study of 53 [range 4–570]). Of these 83 studies, 72 had a unique article, and 11 were selected from studies that had multiple eligible articles reporting different CRP effects (see Table S1), but only one was included in the meta-analysis according to the rules described under “data extraction.” The mean age of patients across studies was a median (IQR) of 62.4 y (60.0–65.3 y). The median (IQR) proportion of women in studies was 24.9 (19–29). No studies reported stable angina as the sole initial presentation; the median (IQR) prevalence of previous myocardial infarction was 39% (26–50). The proportion of stable patients was 100% in 14 studies, median (IQR) of 49.8% (27.7%–67.8%) in 24 studies, and not stated in the remainder.

### Quality of Study Reports

**Figure 1:**
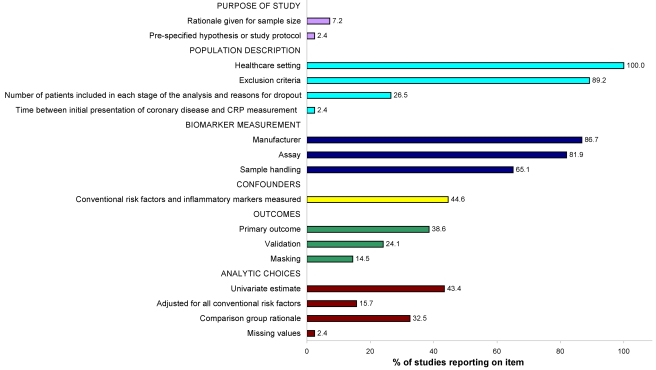
Quality of individual study reports (*n = *17 items, *n = *83 studies), based on the REMARK guidelines [**11**]. Definitions of each item are given in Table S2.

The median (IQR) number of study quality items reported was 7 (6–8) out of a possible 17 and did not change between 1997 and 2009, and was not associated with study size (correlation coefficient of 0.18, *p = *0.11) (Figure 1). More than 80% of studies reported details of the healthcare setting, exclusion criteria, assay type, and manufacturer. Two studies referred to a study protocol, but no studies referred to a statistical analytic protocol. Two studies reported the time elapsed between first lifetime presentation with coronary disease and assessment of CRP. Ten different types of comparisons were used for presenting the relative risks (five based on continuous CRP measures, three with equal sized groups, and two with unequal sized groups [one two-group and one three-group comparison]); the rationale for choosing these groups was stated in 32.5% of studies.

### Meta-analysis Forest Plot

**Figure 2:**
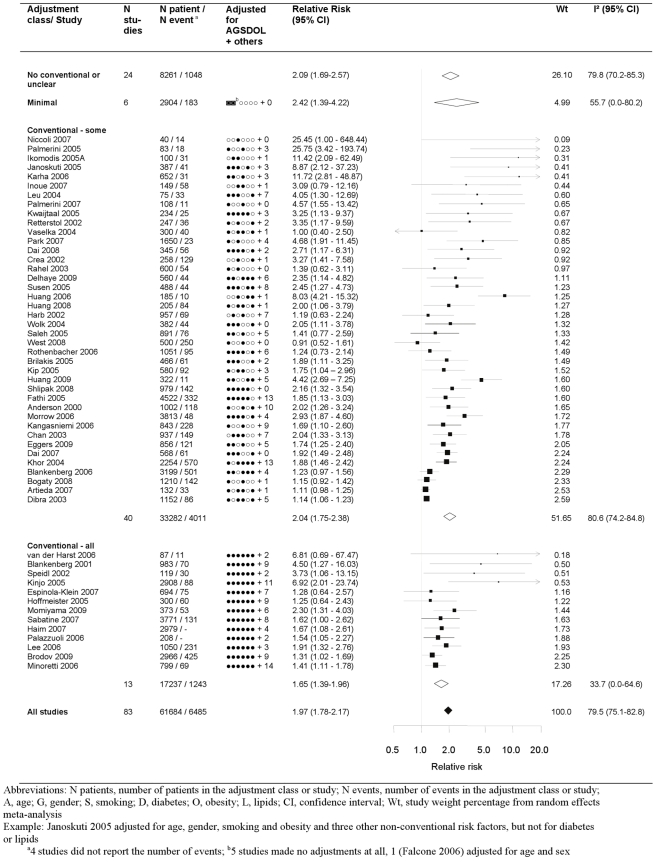
Forest plot of the effect of CRP on prognosis among patients with stable coronary disease. Studies are grouped according to the extent of adjustment for conventional risk factors.

The pooled relative risk from the random effects model of top versus bottom third of CRP using the most highly adjusted study estimate was 1.97 (95% CI 1.78–2.17) (Figure 2). There was marked heterogeneity, with an I^2^ of 79.5% (95% CI 75.1–82.8). Among the 13 studies that adjusted for conventional risk factors (age, sex, smoking, obesity, diabetes, and low-density lipoprotein [LDL] cholesterol), the relative risk was 1.65 (95% CI 1.39–1.96), with a lower I^2^ of 33.7 (95% CI 0.0–64.6). Only three of these studies adjusted, in addition, for fibrinogen or other inflammatory markers and yielded a relative risk of 1.52 (1.25–1.85). The eight studies that adjusted for one or more markers of inflammation, irrespective of adjustment for conventional factors, yielded a relative risk of 1.99 (95% CI 1.45–2.72). Among the 25 studies reporting separate adjustments for age and sex only and for at least one (median 2) conventional risk factor the relative risk for CRP was attenuated by 39%, from 2.44 (95% CI 1.95–3.05) to 1.88 (95% CI 1.55–2.26), respectively. The median (IQR) number of adjustments not including the conventional risk factors was 4 (2–7), encompassing 78 unique risk factors (with hypertension being the most common adjustment variable, appearing in 28 studies).

### Publication Bias

**Figure 3:**
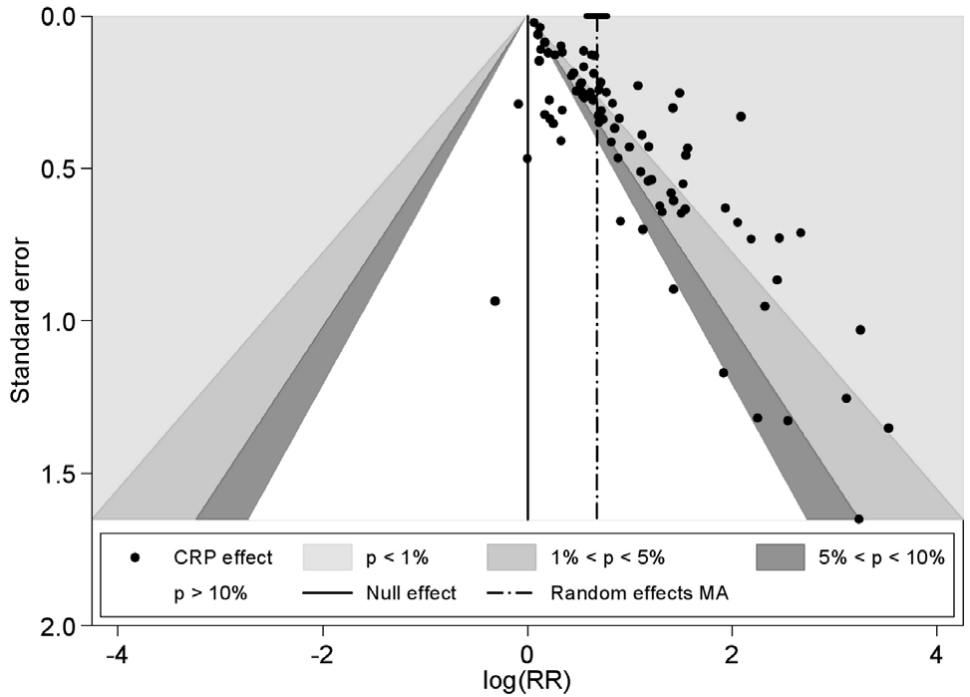
Funnel plot with contours showing different levels of study significance.

The funnel plot was markedly asymmetrical with less precise (smaller) studies reporting higher relative risks than larger studies (Egger's test, *p*<0.001 and Begg's rank correlation test, *p = *0.001) (Figure 3). Adjustment for the extent of publication bias reduced the estimates to between 1.03 (95% CI 0.99–1.07) and 1.63 (95% CI 1.47–1.79), depending on the method used (see Table S2). The quadratic version of the Egger test gave an adjusted estimate for the effect of CRP of 1.19 (95% CI 1.13–1.25). Using this test, there was some evidence that the publication bias was greater for studies reporting multivariate adjustments compared to those reporting only a minimally adjusted estimate (test for interaction, *p*<0.0001).

### Meta-regression

**Figure 4:**
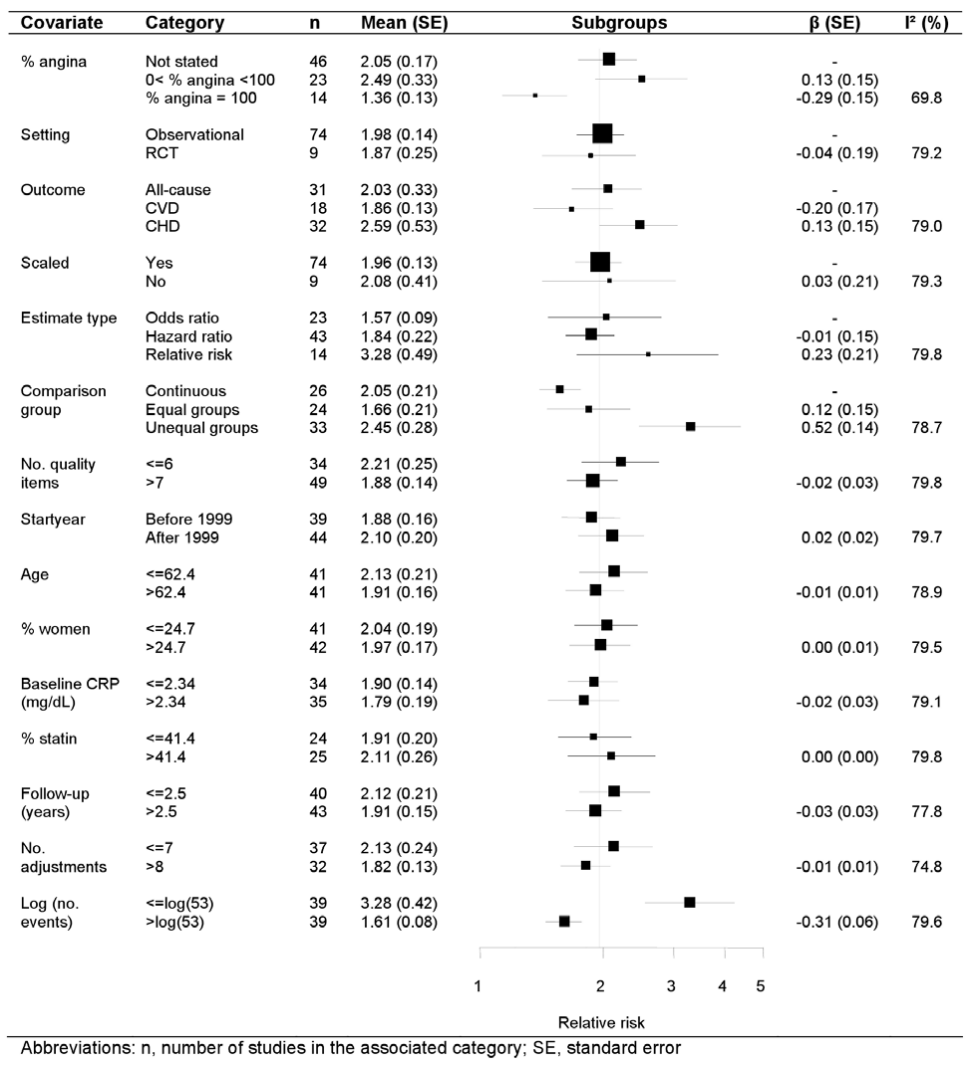
Meta-regression of categorical and continuous study level covariates.

Univariate random effects meta-regression analyses identified four study-level covariates that were associated with the pooled relative risk: definition of comparison group, the number of adjustment variables, the (log) number of events (*p*<0.01), and the proportion of patients with stable coronary disease (*p = *0.02) (Figure 4). Studies originally reporting unequal CRP groups reported stronger effects than those reporting CRP on a continuous scale. Studies reported a median (IQR) of 6 (4–10) adjustment variables, and for each additional adjustment variable the relative risk decreased by 3%. The relative risk was 1.61 among studies with more than the median number of outcome events (*n = *53 events), and 3.28 for smaller studies. The relative risk was 1.47 among studies confined to stable coronary disease, 2.23 in studies with a median of 48.5% stable patients, and 1.96 in the studies in which this proportion was not stated. There was no evidence that the CRP effect differed according to other continuous study level covariates (age, percent women, CRP level, percent on statins, follow-up duration, study start year, number of quality items reported) or to the categorical covariates (event type, type of relative risk). For presentation purposes the meta-regression forest plot is displayed for subgroups, with groups subsequently analysed in the meta-regression chosen for the categorical covariates and continuous covariates split above and below their respective median values. The regression coefficient, associated standard error and the I^2^ value, however, were obtained from random effects meta-regression. The substantial heterogeneity in the meta-analysis remained largely unchanged in the meta-regression, reflected in an I^2^ that stayed at around 80% and a stable random effect variance.

### Discrimination

**Figure 5:**
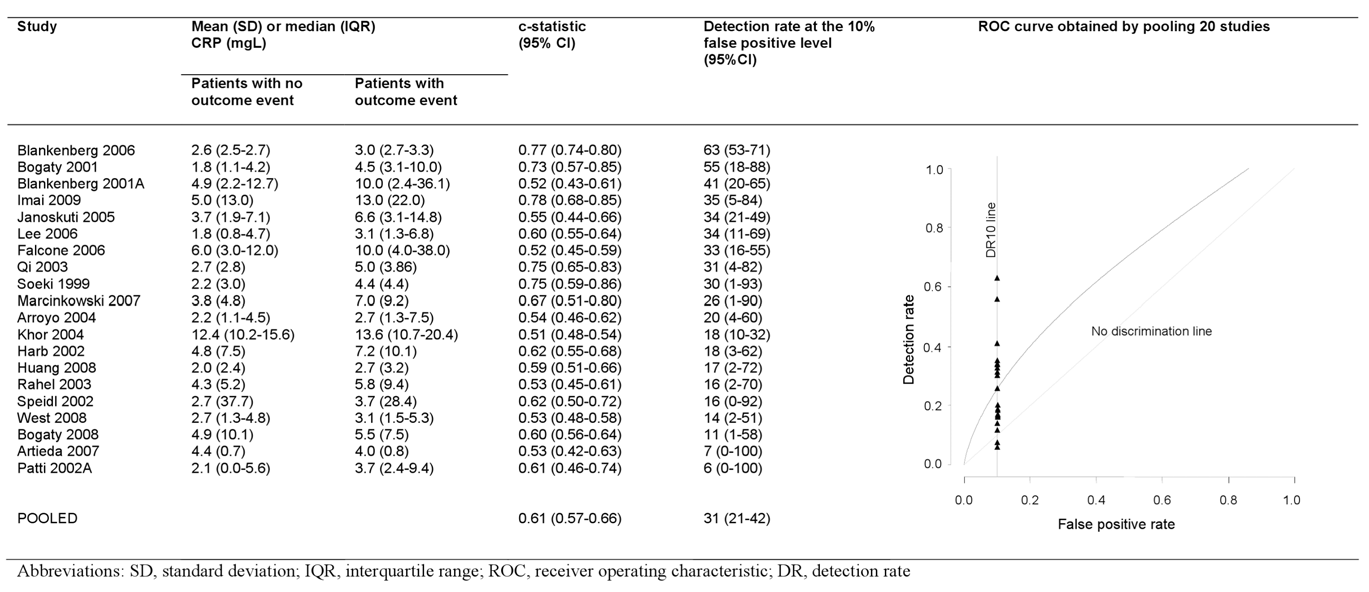
Detection rates at 10% false positive rate and c-statistic for individual studies, and pooled ROC curve.

Only two studies reported the area under the ROC curve or equivalent c-statistic (Figure 5). Nineteen studies reported average CRP values separately among those with and without events enabling calculation of discrimination performance. We found that selecting the cut-off value of CRP that gives a 10% false positive rate (1-specificity), gave a detection rate (sensitivity) of 31% (range 6%–63%) when CRP was used alone as a screening test. Our conclusions on discrimination were based on 20 studies (2,374 events); however, the fact that these did not differ from the other studies in terms of their reported relative risks, (*p = *0.49), and mean number of patients (697 versus 758, *p = *0.97), and that the findings were in line with those reported for aetiologic studies, suggests that these findings are likely to be representative.

## Discussion

In one of the largest (83 studies reporting over 61,000 patients) and most detailed, to our knowledge, evaluations of a single prognostic biomarker, we found the absence of prespecified statistical analytic protocols, publication bias so marked that it could potentially explain much of the association, and multiple types of reporting biases. These biases preclude firm conclusions about the magnitude and independence of the association between higher CRP levels and higher risk of subsequent death and nonfatal cardiovascular events. Taken together with evidence of biases in prognostic biomarker research in cancer [4],[5],[29], stroke [30], trauma [31], and musculoskeletal disorders [32],[33], there is a case for changing the way this type of research is designed and reported.

### Quality of Reporting of Primary Studies

Arguably the most fundamental concern was that 0 studies referred to a prespecified statistical analytic protocol. Indeed only two studies referred to any kind of protocol. Thus it is difficult to know what the specific research objectives were at the start of cohort recruitment, at the time of CRP measurement, or at the onset of the statistical analysis. The rationale for comparison group definition, confounder selection, and other analytic choices, even when stated, may have been made after comparing the results of different analytic approaches. Choosing which results to select for presentation may introduce a bias towards “positive” findings.

Descriptions of study populations in the included studies were poor and potentially biased. Only two studies reported the “stage” of the disease, here operationalised as the duration since initial presentation [34]. Although all studies included patients with stable coronary disease, the magnitude of association between CRP and outcomes was greater among studies in which the percentage of stable patients was not stated.

There are no agreed comprehensive criteria for measuring the quality of study reports. The REMARK reporting guidelines for tumour prognostic markers provide a useful start, but are not currently in a form that lend themselves to measurement, and omit reference to statistical analytic protocols. We operationalised the REMARK guidelines and found that studies reported an average of seven of 17 quality items [12]. There was no increase in the average number of items reported over the 13 y since the first publication. Previous systematic reviews have assessed a smaller set of reporting items (seven items [3], three items [35]). In a systematic review of 117 studies of one prognostic biomarker, P53 in bladder cancer, only 34 studies reported sufficient data to be included in a meta-analysis [5].

### Independence of CRP Effect

We graphically depict the incomplete approach to confounder adjustment. Only 13 studies adjusted for a basic set of conventional risk markers and only eight studies adjusted for fibrinogen or other measure of inflammation. Thus the available evidence does not systematically evaluate the independence of the CRP prognosis association from potential confounders, and the extent of residual confounding is not known. Such adjustments are likely to be important because: first, attenuation of the relative risk between minimally adjusted and adjusted models was about 39% in the 25 studies reporting this comparison. Second, studies including a higher number of adjustment variables reported lower relative risks, with each additional variable being associated with about a 3% reduction of the relative risk.

### Publication Bias

Not only did we find strong evidence that publication bias was operating (most studies were small with a median of 53 events per study, and smaller studies were more likely to report higher relative risks), but we quantified the possible magnitude and impact of this bias. We have previously identified through simulation studies [25], and empirical [15] studies—where a gold standard of unpublished data is available—a method for adjusting for publication bias that outperforms others. This method, a quadratic version of the Egger regression test, attenuated the effect of CRP by 81%. However, all methods of adjustment produced attenuated results, with levels of attenuation ranging from 28% to 96%. It is worth noting that the funnel plot asymmetry is present even for larger studies. The degree of the bias arising from nonpublication calls into question the strength of any association between CRP and outcome.

### Discrimination and Prediction

American Heart Association guidelines [16] recommend reporting measures of discrimination but only two studies in our review did. This reporting of risk prediction is of wide clinical interest because stable coronary disease has a high annual risk of fatal and nonfatal acute coronary syndromes of between 2% [36] and 5% [37],[38] and affects an increasing number of people [39] as the population ages and survival from acute coronary syndromes improves.

Because of the lack of published protocols, we do not know whether other studies carried out, but elected not to report, such analyses. Based on the 20 studies reporting CRP distributions among those with and without events, CRP on its own detected only 31% of patients who would subsequently experience events at a 10% false positive rate. We found a c-statistic of 0.61, similar to the 0.65 observed in healthy population studies [6]. Given the magnitude of the CRP relative risk, and that CRP is correlated with some of the factors (e.g. white cell count, glucose) in existing scores, it seems unlikely that CRP would add substantially to the discrimination achieved by standard clinical factors among patients with stable coronary disease [40],[41]. Even if it does add predictive information, CRP may not be cost-effective [7],[42].

### Comparison with Healthy Population Studies

By contrast with the evidence among patients with coronary disease, the quality of evidence in healthy populations (aetiologic) [6],[19],[43]–[47] is not subject to the same concerns. Sufficiently unbiased and precise estimates of CRP effect have been obtained that allow assessment of confounding in mendelian randomisation approaches, which in turn have questioned the role of CRP in disease onset. A causal role in disease progression is still possible for CRP if, for example, it were associated with thrombosis and necrosis, rather than the development of atherosclerosis. The populations in our systematic review, compared to healthy population studies [6], evaluated the role of higher initial CRP levels (2.3 versus 1.28 mg/l), shorter follow up periods (median 2.5 y versus 3–20 y). and higher annual risk of events (5.5% versus <1%). Observational studies of other markers, such as body mass index are known to exhibit different aetiologic and prognostic effects [48].

### Limitations

The main limitation is that we studied what authors and journal editors select for reporting and not study quality per se. However in many instances it is likely that there is a strong correlation. It is also possible that we missed published studies, although we suspect that the higher quality studies would be more likely to be detected.

### Research Implications

We previously outlined ten steps for improving prognosis research [1], which include the need for prospective study registration, publication of design and analytic protocols, and prospective individual participant data meta-analysis. Pooling data from a subset of larger, higher quality, more homogeneous studies in order to make better adjustments for confounders and further investigate discrimination (e.g., with net reclassification improvement measures) is feasible in such clinical datasets [49]. But our review suggests that identifying such a subset of studies may not be easy, and there is a need for new clinical cohorts. Better reporting is required and the existing guidelines are a start [12],[50], but these require development across disease areas and formalisation into data extraction tools. The CRP–prognosis literature may be summarised as early (phase 1) stage, in which investigators aim to discover and report possible associations [51]. There is a need to move to phase 2 in which these associations are more rigorously evaluated. Such better quality observational evidence is an important basis for prioritizing other methods of addressing confounding [52] such as “mendelian randomisation” [13],[43]–[47] and randomisation to specific biomarker lowering agents [53].

### Clinical Implications

Our findings suggest that clinical guidelines [8],[9] should change their recommendations. The available evidence supports a negative recommendation, i.e., that CRP should not be routinely measured among patients with stable coronary disease to quantify prognosis or to guide interventional therapies. Our findings explicitly challenge the statement for healthcare professionals made by the Centers for Disease Control that measuring CRP is both “useful” and “independent” as a marker of prognosis.

Furthermore, there is a need for a clear framework in which guideline developers can evaluate the type and quality of evidence necessary to make clinical practice recommendations on prognostic biomarkers.

### Conclusion

The quality of published evidence on CRP and prognosis in stable coronary disease is poor and is not sufficient to recommend routine measurement of this biomarker. This review, and others in cancer, constitutes an indictment of the research culture in prognostic biomarkers, and highlights areas for change, the most fundamental of which is the need to register studies along with their analytic protocols.

## Supporting Information

Figure S1 Review and selection of articles.
(0.25 MB TIF)

Table S1 Systematic review of 83 studies reporting effect of CRP on coronary events among patients with stable coronary disease, ordered according to number of events accrued.
(0.80 MB RTF)

Table S2 Definitions of 17 items of study reporting quality.
(0.06 MB RTF)

Table S3 Adjusting for publication bias using different methods as previously described [25].
(0.06 MB RTF)

Text S1 Checklist summarising compliance with MOOSE guidelines.
(0.06 MB DOC)

## Abbreviations

CI: confidence interval
CRP: C-reactive protein
IQR: interquartile
ROC: receiver operating characteristic
