# Supplementary figures and images for: Evaluating the Quality of Research into a Single Prognostic Biomarker: A Systematic Review and Meta-analysis of 83 Studies of C-Reactive Protein in Stable Coronary Artery Disease
Source: PLoS Med. 2010 Jun 1;7(6):e1000286. doi: 10.1371/journal.pmed.1000286 (PMC2879408; doi:10.1371/journal.pmed.1000286)

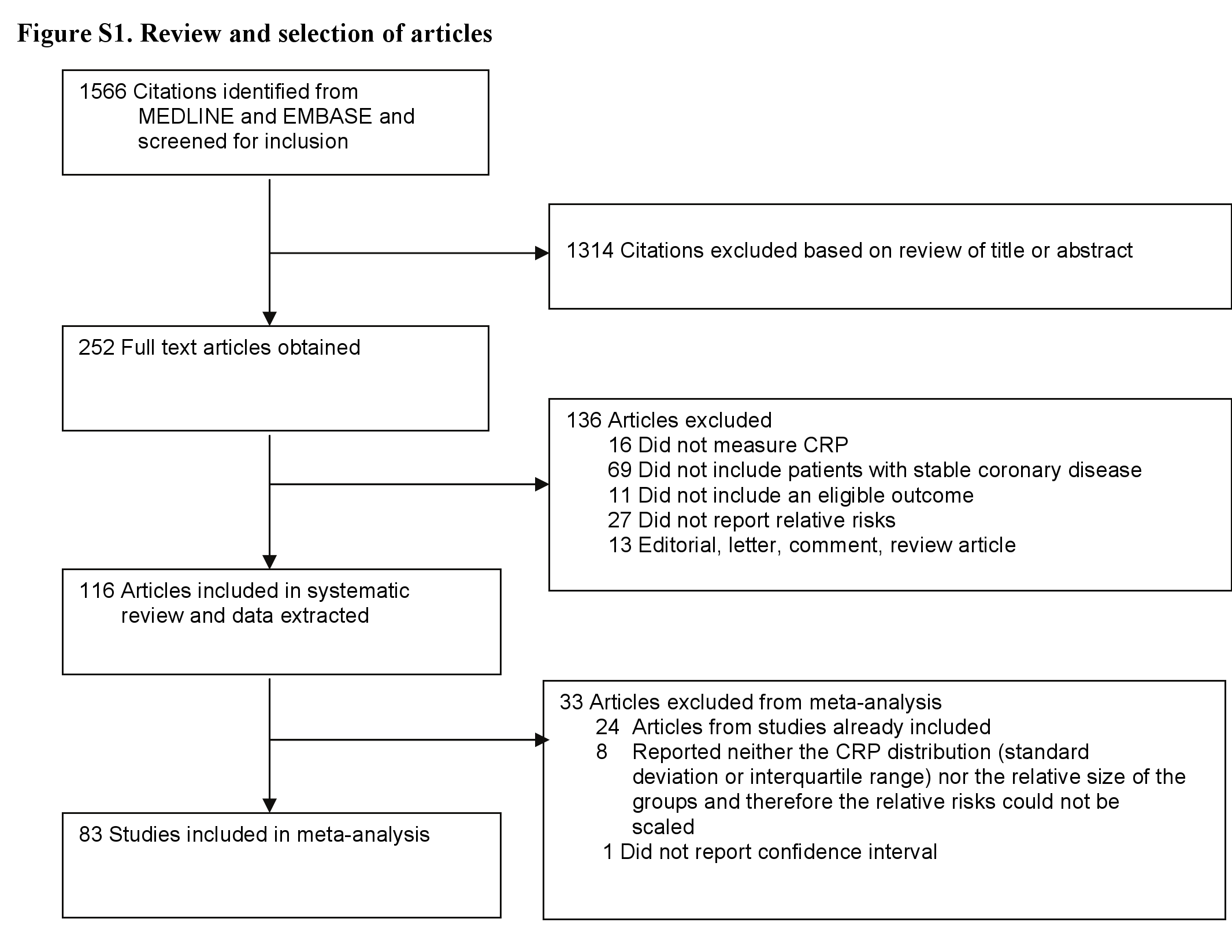

Supplement: Figure S1 — Review and selection of articles. (0.25 MB TIF) [file pmed.1000286.s001.tif]
