# Supplementary material for: Evaluating the Quality of Research into a Single Prognostic Biomarker: A Systematic Review and Meta-analysis of 83 Studies of C-Reactive Protein in Stable Coronary Artery Disease
Source: PLoS Med. 2010 Jun 1;7(6):e1000286. doi: 10.1371/journal.pmed.1000286 (PMC2879408; doi:10.1371/journal.pmed.1000286)
Supplement: Table S1 — Systematic review of 83 studies reporting effect of CRP on coronary events among patients with stable coronary disease, ordered according to number of events accrued. (0.80 MB RTF) [file pmed.1000286.s002.rtf]

Table S1. Systematic review of 83 studies reporting effect of CRP on coronary events among patients with stable coronary disease, ordered according to number of events accrued
Author publication year 
	N patients	Age	% Women	Baseline coronary morbidity: Stable angina/ Angiographic CAD /Prior MI (%)	CRP mean (SD) or median (IQR) (mg/L)	Assay type	Follow up (years)	Event combination	N events	Crude annual risk	Comparison group definition

Group 1	


Group 2	


Group 3	


Group 4	RR	95% CI	
Khor2004†[1-5]
	2254	66	23.8	-/100/20	- 	FP	3.1	ACM	570	8.16	<1.2	≥1.2			1.6d	1.3-1.9	
Blankenberg
2006[6] 
	3199	65.4	23.2	-/100/-	2.65 (0.26)*	N	4.5	CVD	501	3.48	Continuous (per SD)				1.1d
	0.99-1.23	
Brodov2009
[7]	2966	60.2	8.6	-/-/-	0.58 (0.051)* 	CL	6.2	CHD	425	2.31	<2.32	2.32-5.37	>5.37		1.31	1.02-1.69	
Fathi2005[8] 	4522	65	29.1	-/100/42.4	- 	T	1.7	ACM	332	4.32	<1.0	1.0-3.0	>3.0		1.85d	1.13-3.03	
West2008[9] 	500	63	15	-/100/-	- 	LPE	2.5	CVD	250	20	-	-			0.9e
	0.40-1.50	
Lee2006[10] 	1050	60.8	27.1	-/-/-/	-	CL	8.5	ACM	231	2.59
	≤0.88	0.88-1.97	1.97-5.16	≥5.16	2.12e
	1.38-3.27	
Chan2003[11]	937	69.5	31.1	-/100/28.4	4.0a (-)*	-	1	ACM	149	15.9	Continuous (per quartile)

				1.32d	1.12-1.56	
Bogaty2008[12] 	1210	62	25	37/-/28	4.97 (9.83)*	N	1	ACM	142	11.74	Continuous (log transformed)				1.12e
	0.93-1.34	
Shlipak2008
[13] 	979	67	18	-/100/53.7	- 	T	3.7	CHD	142	3.92	<=4.93	>4.93			1.82d	1.24-2.67	
Sabatine
2007[14]
	3771	63.7	18.9	-/-/56.1	1.71 (0.83-3.50)	T	4.8	CVD	131	0.724	<1.0	1.0-3.0	>3.0		1.67d	1.00-2.78	
Crea2002[15] 	258	61.9	10.9	-/-/-/	- 	N	5	ACM	129	10.00	<4.1	≥4.1			2.51e
	1.3-4.8	
Eggers2009[16]	856	67.3	29.1	-/-/82.5	2.3 (1.2-4.4)	-	5	CHD	121	2.83	Continuous (log transformed)				1.3	1.1-1.5	
Kangasniemi
2006[17]
	843	60.6	21.2	-/-/9.5	- 	T	12	CHD	119	1.18	<1.0	≥1.0			1.65f	0.95-2.88	
Anderson2000
[18] 	1002	64.9	22.7	-/100/-	2.34a (-)
	 FP	3.0	ACM	118	3.93	Continuous (per tertile)				1.42d	1.12-.80	
Inaguma2007
[19] 
	790	67.7	27.1	-/-/64.1	3.2 (5.4)	-	2.31	CVD	110	6.03	Continuous (per mg/dL)				1.05d	1.02-1.08	
Falcone2006 [20] 	1014	64.6	27.2	82.9/100/44.9	0.6 (0.3-1.3)	-	2.7	CVD	105	3.84	Continuous (per SD)				1.50d	1.21-1.69	
Fang2007[21] 	258	58.7	36.8	26.7/100/-	1.87a (-)	-	1.01	CHD	102	39.14	<2.64	>2.64			2.0d	0.9-6.7	
Rothenbacher
2006[22] 	1051	58.5	15.1	-/100/58.2	- 	LPE	4.1	CVD	95	2.20	≤1.24	1.25-3.51	3.52-8.61	>8.61	1.29d 	0.69-2.43	
Kip2005[23] 


	580	58	100	-/61/-	2.93 (1.68-5.28) 	-	4.7	CVD	92	3.37	<0.17	0.17-0.36	0.37-0.83	≥0.84	1.92d	1.04-3.54	
Garcia-Moll2000[24] 	911	63.1	35.9	100/23.9/31.7	4.01 (5.99)*
	-	1.6	CVD	89	6.11	Continuous (Log10)				1.68e 	1.04-2.72	
Otsuka2002[25]
	363	65.3	29.5	-/100/27.5	0.39 (0.75)*	LXAG	0.54	CVD	89	45.4	Continuous (per mg/dL)				1.14e
	0.82-1.58	
Dibra2003[26]	1152	66.1	26.6	100/100/31.5	-	-	1	ACM	86	7.47	≤5	>5			1.8d
		
Ndrepepa2006†
[27,28]	989	66.3	21.0	-/100/39.9	7.79 (16.35)*	T	3.6	ACM	85	2.39	<1.2	≥1.2			2.3e
	1.40-3.78	
Huang2008[29]	205	68	11.5	-/62.9/0	2.3 (2.8)	N	4	Morbidity	84	10.24	<1.1	>=1.1			1.66f
	1.04-2.64	
Saleh2005†
[30,31] 	891	63.6	27.0	68/100/43	2.25 (1.1-4.8)	N	2.6	ACM	76	3.28	≤1.0	1.1-3.1	>3.2		1.41e	0.77-2.60
	
Espinola-Klein2007[32] 	694	62.4	27.4	-/92.1/43.3	4.8a (-) 	LPE	6.5	CVD	75	1.66	<4.8	>=4.8			1.2d 	0.8-2.2	
Chew2001[33]	727	65.9	28.6	-/100/30.9	5.0a (-) 	-	0.082	ACM	71	119.1	<0.16	0.16-0.40	0.41-1.10	>1.10	3.68e
	1.51-8.99	
Blankenberg
2001†[34–38,38-41]	983	62.2	26.4	78.4/100/51.8	 -	LPE	3.1	CHD	70	2.30	<9.6	≥9.6			3.10d
	1.2-8.1	
Harb2002[42]
	957	-	24.6	32.2/-/100	- 	-	2.17	CHD	69	3.32	≤0.09
	0.10-0.23	0.24-0.58	>0.59	1.22d	0.58-2.55	
Minoretti
2006[43] 	799	64.9	25.6	100/100/46.3	0.5 (0.3-1.2)b*	 -	2.7	CVD	69	3.20	Continuous (log mg/dL)				1.42d	1.12-1.81	
Arroyo- Espliguero 2004†[44,45]	700	63	25.0	100/-/39	2.3 (1.1-4.6) 	-	1.0	CHD	68	9.71	Continuous (log mg/L)				1.9e
	1.1-3.5	
Ijsselmuiden
2003[46] 	400	60.7	19	67.5/100/38.0	- 	-	0.5	CVD	64	32.0	≤10.0	>10.0			1.94e
	1.0-3.7	
Dai2007[47]	568	62.5	33.8	100/100/-	7.75 (30.31)*	N	1.85	CVD	61	5.8	Continuous (log transformed)				1.51d	1.28-1.77	
Brilakis2005
[48] 	466	60.1	38	-/75.8/15	2.9 (1.2-6.7) 	T	4	ACM	61	3.27	Continuous (per 1.32mg/dL)				1.34d	1.05-1.72	
Hoffmeister
2005[49] 	300	57.9	14.4	-/100/61.3	-	N	3.2	CVD	60	6.25	<0.69	0.70-1.27	1.28-2.84	>2.85	1.3d	0.6-2.8	
Inoue2007†
[50,51] 	149	63	29	53.7/83.2/29.5	2.1 (8.6) 	LPE	7	CVD	58	5.56	-	-			2.28f	0.92-6.81	
Dai2008[52]	345	64.6	26.7	-/100/15	8.40 (25.7)*	N	3	CHD 	56	5.41	Continuous (-)				1.99d	1.11-3.56	
DeWinter2003† [53-55] 	1458	61.5	27.6	-/100/-	6.64 (11.23)*	N	1.16	CHD	55	3.25	≤3	>3			4.50e
	1.70-11.9	
Rahel2003[56]
	600	61.6	31.3	-/100/-	4.47 (5.67)*	ELISA	0.67	ACM	54	13.4	-	-	-		1.39e
	0.62-3.10	
Momiyama2009[57]	373	64	21	-/72/22	0.7c (-)	N	2.9	CVD	53	4.90	<=1.0	>1.0			2.00	1.1-3.4	
Wu2005[58]	150	67.8	9.3	100/100/19.7	- 	-	1.5	CHD	48	21.3	<1.0	≥1.0			1.91d	0.98-3.74	
Delhaye2009
[59]	560	61.3	22.5	-/-/-/	3.1 (1.36-8.63)	N	1.04	CHD	44	7.55	Continuous (log transformed)				1.33	1.05-1.70	
Susen2005[60]
	488	61.0	22.0	69.0/100/19.0	2.60 (1.20-7.09)*	N	1.24	CHD	44	7.27	Continuous (per unit by log transformation)				2.05d	1.21-3.47	
Wolk2004[61] 	382	62.0	30.0	-/100/20	1.12 (1.33) 	LPE	4	CVD	44	2.88	Continuous (per SD)				1.39d	1.05-.84	
Janoskuti
2005[62] 	387	59	26.9	-/-/48.1	3.89 (1.92-7.47) 	N	5.06	ACM	41	2.09	<6.24	≥6.24			5.21d 	1.76-5.43	
Veselka2005
[63] 	300	63.5	31.0	100/99.5/57.0	- 	N	0.5	ACM	40	26.7	≤3.0	>3.0			1.00e
	0.51-1.95	
Low2004[64] 	347	58	34.6	69.2/-/16.5	- 	IPA	2.5	CVD	37	4.27	<1.0	≥1.0			3.47f
	1.76-6.84	
Retterstol
2002[65] 	247	52.7	21.9	-/-/100	2.36 (1.19-4.19) 	LPE	10	CHD	36	1.46	≤1.19	1.20-2.36	2.37-4.19	≥4.20	4.09f
	1.20-3.93	
Gach2007[66] 	89	60.2	24.7	100/100/25.8	3.35 (5.1)  	N	6.6	CHD	36	6.13	<3.0	≥3.0			1.05d	1.02-.09	
Artieda2007
[67] 	132	55.2	0	100/72.7/0	0.45 (0.06)*	N	3.98	CVD	33	6.28	Continuous (log mg/dL)				2.17f 
	0.87-5.43	
Imai2009[68]	111	71	79.9	63.9/-/0	0.72 (1.57)*	N	1.9	CHD	33	15.6	-	-	-	-	1.39	1.06-1.81	
Leu2004[69] 	75	68.1	12	100/100/25.3	1.02 (0.20-0.99)c 	ELISA	3.33	CVD
	33	13.2	≤1.0	>1.0			2.78f
	1.21-6.41	
Qi2003†
[70,71]	134	64.1	19.4	48.5/100/34.4	3.27 (3.21)*	EIA	1	CHD	32	23.9	Continuous (unit increase not specified)				2.03f
	1.13-2.05	
Karha2006[72]
	652	65.4	32.4	-/-/-/	3.3 (1.7-9.7) 	T	1	ACM	31	4.75	<3.3	≥3.3			6.5e	2.2-19.3	
Ikonomides
2005[73]
	100	54	16	100/100/52	- 	N	6	CHD	31	5.17	<2.4	≥2.5			6.24d	1.74-22.42	
Speidl2002[74] 	119	39.3	23.5	-/100/78.2	-	-	4.5	CHD	30	5.60	<1.59	1.69-5.51	>5.58		3.73e 
	1.06-13.18	
Haverkate1997†
[75,76] 	743	56	14.1	100/-/42	1.71 (0.2-15.4)b*	EIA	2	CHD	29	1.95	Continuous (per SD)				1.5f
	1.01-2.18	
Kubica2005†
[77]	80	56.0	27.5	87.5/100/50	1.2a (-) 	N	1	CHD	28	35.0	<0.85	0.85-2.0	>2.00		4.17e

	1.27-13.65	
Kwaijtaal2005 [78,79]
	213	53.6	21.8	9.9/100/26.8	3.69 (5.0) 	ELISA	2	CHD	25	5.34	≤3.0	>3.0			2.50f 	1.1-5.7	
Park2007[80]	1650	60.3	28.7	53.7/100/8.0	- 	LPE	1	CHD	23	1.39	<1.2	1.2-3.1	>3.1		9.94d	1.28-77.14	
Bogaty2001
[81] 	50	57.9	12 	-/100/0	3.0 (5.2) 	N	4	Morbidity	23	5.75	Continuous (per mg/dL)				5.4e  	1.9-17.2 	
Zairis2002[82] 	483	59.3	18.0	22.2/100/8.7	5.8 (5.0-51.0)c 	T	3	CHD	20	1.38	<0.68	≥0.68			3.16d	1.25-7.98	
Aytekin2003
[83]	116	56.5	22.4	34.5/100/14.7	-	-	0.5	CHD	19	32.8	≤0.5	>0.5			2.58d	1.05-6.31	
Palmerini[84]
2005	83	72.0	40.0	25.0/100/33.0	- 	N	0.75	ACM	18	28.9	<10.36	≥10.36			11.5e
 	2.5-52	
Grander2004
[85]
	81	61.6	30.9	-/100/-	4.3 (0.3-48.2) 	-	0.57	CVD	17	36.82	0.7-4.8	0.23-0.69	≤0.22		0.045e

	0.004-0.522
	
Marcinkowski
2007[86] 	107	58.3	22.4	-/-/0	4.44 (6.07)* 	N	1.48	CHD	15	10.14	<=1.83	>1.83			14.39d	1.94-106.7	
Kinjo2005†
[87,88] 	1191	62.4	25.9	-/-/15.4	9.20 (12.0)* 	N	1	ACM	14	1.18	<2.9	≥2.9			1.28d
	0.21-7.23	
Lu2003[89] 	153	71.0	13.1	100/100/30.1	-
	N	1.33	CVD	14	6.88	≤0.5	>0.5			0.77f
	0.17-3.47
	
Niccoli2007
[90] 	40	61	15	35/-/-	2.7 (1.5-4.6)*	N	0.5	ACM	14	70	<=3	>3			10.9e
	1.0-119	
Biancari2003
[91] 	764	64	24.9	-/100/43.7	- 	-	0.014a	ACM	13	121.5	<1.00	≥1.00			6.97e
	1.45-33.42	
Gaspardone
1998[92] 	76	58.7	14.5	100/100/-	2.3 (1.9-2.9) 	T	1	CHD	13	17.11	≤0.5
	>0.5			5.54f	0.77-39.9 	
Patti2002[93] 	73	6.0	15.0	51.0/100/55.0	2.5 (0.1-5.9) 	N	1.5	CHD	12	11.0	-	-	-	-	5.28e
	0.68-40.92
	
Huang2009
[94]	322	62.1	26.1	-/-/20.8	2.33 (0.32)*	N	2	CHD	11		<=3.0	>3.0			3.26 	2.20-4.84	
Palmerini2007
[95] 	108	69.1	23	28.7/100/32.5	-	N	0.75	ACM	11	13.58	<1.22	>=1.22			5.87d
	1.67-20.62	
Soeki1999[96] 	106	62.3	25.5	-/-/35.8	2.43 (3.22)*	LXAG	4.17	CHD	11	2.49	Continuous (per SD)				1.55d	1.08-2.23	
van der Harst
2006[97]	87	62.9	14.9	7.4/100/41.4	1.9 (0.9-4.7) 	ELISA	7.6	CVD	11	1.66	≤1.9	>1.9			4.3d	0.75-24.55
	
Huang2006
[98]
	185	69.4	47	-/100/-	-	ELISA	3	CVD	10	1.80	≤3.0	>3.0			4.6d
	2.51-6.47	
Milazzo1999
[99] 	86	64.7	14.3	17.4/100/47.7	- 	N	3.2	ACM	4	1.45	<3.0	≥3.0			6.86f 	0.96-49.3	
Morrow2006
[100] 	3817	60.7	24.1	-/-/39	2.4 (1.2-5.5) 	T	2	CVD	4	-	<1.0	1.0-3.0	>3.0		3.9d
	1.8-5.6	
Haim2007[101] 	2979	60	8.6	57.3/-/78	-	CL	6.2	CHD	-	-	Continuous (per natural log unit)				1.28d
	1.04-1.59	
Palazzuoli
2006[102] 	208	70.6	33.7	21.2/-/31.7	-	N	1	Morbidity	-	-	≤5.0	>5.0			1.4e
	1.14-2.08	
Schaan2007
[103] 	123	58.2	48.9	-/37.8/100	5.83 (1.39)* 	N	2.27	CHD	-	-	Continuous (per mg/dL)				1.059f
	1.00-1.12	
Chirinos2005
[104] 	122	63.9	0	-/100/39	0.68 (0.18-1.54)	N	3	ACM	-	-	Continuous (per mg/dL)				1.26e
	1.02-1.55	
* Calculated from reported values
† Article chosen where two or more articles reporting different CRP effects were reported 
aMean bGeometric mean (95% confidence interval) cMedian (Range) 	dHazard ratio eOdds ratio fRelative risk
FP Fluorescence polarisation; N Nephelometry; T Turbidimetric; LPE Latex particle enhanced ; CL Chemiluminescence; LXAG Latex agglutination; ELISA Enzyme linked immunoradiometric assay; IPA Infrared particle assay; EIA Enzyme immunoassay
ACM All-cause mortality; CVD Fatal or non-fatal cardiovascular events; CHD Fatal or non-fatal coronary events


Reference List

	1. 	Khor LL, Muhlestein JB, Carlquist JF, Horne BD, Bair TL, et al. (2004) Sex- and age-related differences in the prognostic value of C-reactive protein in patients with angiographic coronary artery disease. Am J Med 117: 657-664.
	2. 	Muhlestein JB, Horne BD, Carlquist JF, Madsen TE, Bair TL, et al. (2000) Cytomegalovirus seropositivity and C-reactive protein have independent and combined predictive value for mortality in patients with angiographically demonstrated coronary artery disease. Circulation 102: 1917-1923.
	3. 	Muhlestein JB, Anderson JL, Horne BD, Carlquist JF, Bair TL, et al. (2004) Early effects of statins in patients with coronary artery disease and high C-reactive protein. Am J Cardiol 94: 1107-1112.
	4. 	Horne BD, Muhlestein JB, Carlquist JF, Bair TL, Madsen TE, et al. (2000) Statin therapy, lipid levels, C-reactive protein and the survival of patients with angiographically severe coronary artery disease. J Am Coll Cardiol 36: 1774-1780.
	5. 	Zebrack JS, Muhlestein JB, Horne BD, Anderson JL (2002) C-reactive protein and angiographic coronary artery disease: independent and additive predictors of risk in subjects with angina. J Am Coll Cardiol 39: 632-637.
	6 	Blankenberg S, McQueen MJ, Smieja M, Pogue J, Balion C, et al. (2006) Comparative impact of multiple biomarkers and N-Terminal pro-brain natriuretic peptide in the context of conventional risk factors for the prediction of recurrent cardiovascular events in the Heart Outcomes Prevention Evaluation (HOPE) Study. Circulation 114: 201-208.
	7. 	Brodov Y, Behar S, Goldenberg I, Boyko V, Chouraqui P (2009) Usefulness of combining serum uric acid and C-reactive protein for risk stratification of patients with coronary artery disease (Bezafibrate Infarction Prevention [BIP] study). Am J Cardiol 104: 194-198.
	8. 	Fathi RB, Gurm HS, Chew DP, Gupta R, Bhatt DL, et al. (2005) The interaction of vascular inflammation and chronic kidney disease for the prediction of long-term death after percutaneous coronary intervention. Am Heart J 150: 1190-1197.
	9. 	West MJ, Nestel PJ, Kirby AC, Schnabel R, Sullivan D, et al. (2008) The value of N-terminal fragment of brain natriuretic peptide and tissue inhibitor of metalloproteinase-1 levels as predictors of cardiovascular outcome in the LIPID study. Eur Heart J 29: 923-931.
	10. 	Lee KW, Hill JS, Walley KR, Frohlich JJ (2006) Relative value of multiple plasma biomarkers as risk factors for coronary artery disease and death in an angiography cohort. CMAJ 174: 461-466.
	11. 	Chan AW, Bhatt DL, Chew DP, Reginelli J, Schneider JP, et al. (2003) Relation of inflammation and benefit of statins after percutaneous coronary interventions. Circulation 107: 1750-1756.
	12. 	Bogaty P, Boyer L, Simard S, Dauwe F, Dupuis R, et al. (2008) Clinical utility of C-reactive protein measured at admission, hospital discharge, and 1 month later to predict outcome in patients with acute coronary disease. The RISCA (recurrence and inflammation in the acute coronary syndromes) study. J Am Coll Cardiol 51: 2339-2346.
	13. 	Shlipak MG, Ix JH, Bibbins-Domingo K, Lin F, Whooley MA (2008) Biomarkers to predict recurrent cardiovascular disease: the Heart and Soul Study. Am J Med 121: 50-57.
	14. 	Sabatine MS, Morrow DA, Jablonski KA, Rice MM, Warnica JW, et al. (2007) Prognostic significance of the Centers for Disease Control/American Heart Association high-sensitivity C-reactive protein cut points for cardiovascular and other outcomes in patients with stable coronary artery disease. Circulation 115: 1528-1536.
	15. 	Crea F, Monaco C, Lanza GA, Maggi E, Ginnetti F, et al. (2002) Inflammatory predictors of mortality in the Scandinavian Simvastatin Survival Study. Clin Cardiol 25: 461-466.
	16. 	Eggers KM, Lagerqvist B, Venge P, Wallentin L, Lindahl B (2009) Prognostic value of biomarkers during and after non-ST-segment elevation acute coronary syndrome. J Am Coll Cardiol 54: 357-364.
	17. 	Kangasniemi OP, Biancari F, Luukkonen J, Vuorisalo S, Satta J, et al. (2006) Preoperative C-reactive protein is predictive of long-term outcome after coronary artery bypass surgery. Eur J Cardiothorac Surg 29: 983-985.
	18. 	Anderson JL, Muhlestein JB, Horne BD, Carlquist JF, Bair TL, et al. (2000) Plasma homocysteine predicts mortality independently of traditional risk factors and C-reactive protein in patients with angiographically defined coronary artery disease. Circulation 102: 1227-1232.
	19. 	Inaguma D, Tatematsu M, Shinjo H, Suzuki S, Mishima T, et al. (2007) Relationship between renal function at the time of percutaneous coronary intervention and prognosis in ischemic heart disease patients. Clin Exp Nephrol 11: 56-60.
	20. 	Falcone C, Minoretti P, D'Angelo A, Buzzi MP, Coen E, et al. (2006) Markers of eosinophilic inflammation and risk prediction in patients with coronary artery disease. Eur J Clin Invest 36: 211-217.
	21. 	Fang Y, Huang L, Li A, Song Y, Geng Z, et al (2007) Significance of the ratio of circulating endothelial cell expressing endothelial lipase and supersensitivity C-reactive protein in prognosis of patients with coronary arterty disease. Chin Crit Care Med 19: 644-646.
	22. 	Rothenbacher D, Koenig W, Brenner H (2006) Comparison of N-terminal pro-B-natriuretic peptide, C-reactive protein, and creatinine clearance for prognosis in patients with known coronary heart disease. Arch Intern Med 166: 2455-2460.
	23. 	Kip KE, Marroquin OC, Shaw LJ, Arant CB, Wessel TR, et al. (2005) Global inflammation predicts cardiovascular risk in women: a report from the Women's Ischemia Syndrome Evaluation (WISE) study. Am Heart J 150: 900-906.
	24. 	Garcia-Moll X, Zouridakis E, Cole D, Kaski JC (2000) C-reactive protein in patients with chronic stable angina: differences in baseline serum concentration between women and men. Eur Heart J 21: 1598-1606.
	25. 	Otsuka M, Hayashi Y, Ueda H, Imazu M, Kohno N (2002) Predictive value of preprocedural fibrinogen concerning coronary stenting. Atherosclerosis 164: 371-378.
	26. 	Dibra A, Mehilli J, Braun S, Hadamitzky M, Baum H, et al. (2003) Association between C-reactive protein levels and subsequent cardiac events among patients with stable angina treated with coronary artery stenting. Am J Med 114: 715-722.
	27. 	Ndrepepa G, Kastrati A, Braun S, Koch W, Kolling K, et al. (2006) A prospective cohort study of predictive value of homocysteine in patients with type 2 diabetes and coronary artery disease. Clin Chim Acta 373: 70-76.
	28. 	Ndrepepa G, Kastrati A, Braun S, Mehilli J, Niemoller K, et al. (2006) N-terminal probrain natriuretic peptide and C-reactive protein in stable coronary heart disease. Am J Med 119: 355-358.
	29. 	Huang PH, Lu TM, Wu TC, Lin FY, Chen YH, et al. (2008) Usefulness of combined high-sensitive C-reactive protein and N-terminal-probrain natriuretic peptide for predicting cardiovascular events in patients with suspected coronary artery disease. Coron Artery Dis 19: 187-193.
	30. 	Saleh N, Svane B, Hansson LO, Jensen J, Nilsson T, et al. (2005) Response of serum C-reactive protein to percutaneous coronary intervention has prognostic value. Clin Chem 51: 2124-2130.
	31. 	Saleh N, Braunschweig F, Jensen J, Tornvall P (2006) Usefulness of preprocedural serum N-terminal pro-brain natriuretic peptide levels to predict long-term outcome after percutaneous coronary intervention in patients with normal troponin T levels. Am J Cardiol 97: 830-834.
	32. 	Espinola-Klein C, Rupprecht HJ, Bickel C, Lackner K, Schnabel R, et al. (2007) Inflammation, atherosclerotic burden and cardiovascular prognosis. Atherosclerosis 195: e126-e134.
	33. 	Chew DP, Bhatt DL, Robbins MA, Penn MS, Schneider JP, et al. (2001) Incremental prognostic value of elevated baseline C-reactive protein among established markers of risk in percutaneous coronary intervention. Circulation 104: 992-997.
	34. 	Blankenberg S, Rupprecht HJ, Bickel C, Hafner G, Meyer J (2001) [The role of inflammation and infection in acute coronary syndrome]. Herz 26 Suppl 1: 9-18.
	35. 	Bickel C, Rupprecht HJ, Blankenberg S, Espiniola-Klein C, Schlitt A, et al. (2002) Relation of markers of inflammation (C-reactive protein, fibrinogen, von Willebrand factor, and leukocyte count) and statin therapy to long-term mortality in patients with angiographically proven coronary artery disease. Am J Cardiol 89: 901-908.
	36. 	Blankenberg S, Rupprecht HJ, Bickel C, Peetz D, Hafner G, et al. (2001) Circulating cell adhesion molecules and death in patients with coronary artery disease. Circulation 104: 1336-1342.
	37. 	Lubos E, Schnabel R, Rupprecht HJ, Bickel C, Messow CM, et al. (2006) Prognostic value of tissue inhibitor of metalloproteinase-1 for cardiovascular death among patients with cardiovascular disease: results from the AtheroGene study. Eur Heart J 27: 150-156.
	38. 	Schnabel R, Lackner KJ, Rupprecht HJ, Espinola-Klein C, Torzewski M, et al. (2005) Glutathione peroxidase-1 and homocysteine for cardiovascular risk prediction: results from the AtheroGene study. J Am Coll Cardiol 45: 1631-1637.
	39. 	Sinning JM, Bickel C, Messow CM, Schnabel R, Lubos E, et al. (2006) Impact of C-reactive protein and fibrinogen on cardiovascular prognosis in patients with stable angina pectoris: the AtheroGene study. Eur Heart J 27: 2962-2968.
	40. 	Schnabel R, Blankenberg S, Lubos E, Lackner KJ, Rupprecht HJ, et al. (2005) Asymmetric dimethylarginine and the risk of cardiovascular events and death in patients with coronary artery disease: results from the AtheroGene Study. Circ Res 97: e53-e59.
	41. 	Blankenberg S, Rupprecht HJ, Poirier O, Bickel C, Smieja M, et al. (2003) Plasma concentrations and genetic variation of matrix metalloproteinase 9 and prognosis of patients with cardiovascular disease. Circulation 107: 1579-1585.
	42. 	Harb TS, Zareba W, Moss AJ, Ridker PM, Marder VJ, et al. (2002) Association of C-reactive protein and serum amyloid A with recurrent coronary events in stable patients after healing of acute myocardial infarction. Am J Cardiol 89: 216-221.
	43. 	Minoretti P, Falcone C, Calcagnino M, Emanuele E, Buzzi MP, et al. (2006) Prognostic significance of plasma osteopontin levels in patients with chronic stable angina. Eur Heart J 27: 802-807.
	44. 	Arroyo-Espliguero R, Avanzas P, Cosin-Sales J, Aldama G, Pizzi C, et al. (2004) C-reactive protein elevation and disease activity in patients with coronary artery disease. Eur Heart J 25: 401-408.
	45. 	Arroyo-Espliguero R, Avanzas P, Quiles J, Kaski JC (2009) Predictive value of coronary artery stenoses and C-reactive protein levels in patients with stable coronary artery disease. Atherosclerosis 204: 239-243.
	46. 	Ijsselmuiden AJ, Serruys PW, Scholte A, Kiemeneij F, Slagboom T, et al. (2003) Direct coronary stent implantation does not reduce the incidence of in-stent restenosis or major adverse cardiac events: six month results of a randomized trial. Eur Heart J 24: 421-429.
	47. 	Dai DF, Lin JW, Kao JH, Hsu CN, Chiang FT, et al. (2007) The effects of metabolic syndrome versus infectious burden on inflammation, severity of coronary atherosclerosis, and major adverse cardiovascular events. J Clin Endocrinol Metab 92: 2532-2537.
	48. 	Brilakis ES, McConnell JP, Lennon RJ, Elesber AA, Meyer JG, et al. (2005) Association of lipoprotein-associated phospholipase A2 levels with coronary artery disease risk factors, angiographic coronary artery disease, and major adverse events at follow-up. Eur Heart J 26: 137-144.
	49. 	Hoffmeister A, Rothenbacher D, Kunze M, Brenner H, Koenig W (2005) Prognostic value of inflammatory markers alone and in combination with blood lipids in patients with stable coronary artery disease. Eur J Intern Med 16: 47-52.
	50. 	Inoue T, Kotooka N, Morooka T, Komoda H, Uchida T, et al. (2007) High molecular weight adiponectin as a predictor of long-term clinical outcome in patients with coronary artery disease. Am J Cardiol 100: 569-574.
	51. 	Inoue T, Komoda H, Nonaka M, Kameda M, Uchida T, et al. (2008) Interleukin-8 as an independent predictor of long-term clinical outcome in patients with coronary artery disease. Int J Cardiol 124: 319-325.
	52. 	Dai DF, Hwang JJ, Lin JL, Lin JW, Hsu CN, et al. (2008) Joint effects of N-terminal pro-B-type-natriuretic peptide and C-reactive protein vs angiographic severity in predicting major adverse cardiovascular events and clinical restenosis after coronary angioplasty in patients with stable coronary artery disease. Circ J 72: 1316-1323.
	53. 	de Winter RJ, Koch KT, van Straalen JP, Heyde G, Bax M, et al. (2003) C-reactive protein and coronary events following percutaneous coronary angioplasty. Am J Med 115: 85-90.
	54. 	de Winter RJ, Heyde GS, Koch KT, Fischer J, van Straalen JP, et al. (2002) The prognostic value of pre-procedural plasma C-reactive protein in patients undergoing elective coronary angioplasty. Eur Heart J 23: 960-966.
	55. 	de Winter RJ, Stroobants A, Koch KT, Bax M, Schotborgh CE, et al. (2004) Plasma N-terminal pro-B-type natriuretic peptide for prediction of death or nonfatal myocardial infarction following percutaneous coronary intervention. Am J Cardiol 94: 1481-1485.
	56. 	Rahel BM, Visseren FL, Suttorp MJ, Plokker TH, Kelder JC, et al. (2003) Preprocedural serum levels of acute-phase reactants and prognosis after percutaneous coronary intervention. Cardiovasc Res 60: 136-140.
	57. 	Momiyama Y, Kawaguchi A, Kajiwara I, Ohmori R, Okada K, et al. (2009) Prognostic value of plasma high-sensitivity C-reactive protein levels in Japanese patients with stable coronary artery disease: the Japan NCVC-Collaborative Inflammation Cohort (JNIC) Study. Atherosclerosis 207: 272-276.
	58. 	Wu TC, Leu HB, Lin WT, Lin CP, Lin SJ, et al. (2005) Plasma matrix metalloproteinase-3 level is an independent prognostic factor in stable coronary artery disease. Eur J Clin Invest 35: 537-545.
	59. 	Delhaye C, Sudre A, Lemesle G, Marechaux S, Broucqsault D, et al. (2009) Preprocedural high-sensitivity C-reactive protein predicts death or myocardial infarction but not target vessel revascularization or stent thrombosis after percutaneous coronary intervention. Cardiovasc Revasc Med 10: 144-150.
	60. 	Susen S, Sautiere K, Mouquet F, Cuilleret F, Chmait A, et al. (2005) Serum hepatocyte growth factor levels predict long-term clinical outcome after percutaneous coronary revascularization. Eur Heart J 26: 2387-2395.
	61. 	Wolk R, Berger P, Lennon RJ, Brilakis ES, Johnson BD, et al. (2004) Plasma leptin and prognosis in patients with established coronary atherosclerosis. J Am Coll Cardiol 44: 1819-1824.
	62. 	Janoskuti L, Forhecz Z, Hosszufalusi N, Kleiber M, Walentin S, et al. (2005) High levels of C-reactive protein with low total cholesterol concentrations additively predict all-cause mortality in patients with coronary artery disease. Eur J Clin Invest 35: 104-111.
	63. 	Veselka J, Prochazkova S, Duchonova R, Homolova I, Tesar D (2005) Relationship of C-reactive protein to adverse cardiovascular events in patients treated by percutaneous coronary intervention for stable angina pectoris. Int Heart J 46: 195-204.
	64. 	Low AF, Seow SC, Yeoh KG, Lim YT, Tan HC, et al. (2004) High-sensitivity C-reactive protein is predictive of medium-term cardiac outcome in high-risk Asian patients presenting with chest pain syndrome without myocardial infarction. Ann Acad Med Singapore 33: 407-412.
	65. 	Retterstol L, Eikvar L, Bohn M, Bakken A, Erikssen J, et al. (2002) C-reactive protein predicts death in patients with previous premature myocardial infarction--a 10 year follow-up study. Atherosclerosis 160: 433-440.
	66. 	Gach O, Legrand V, Biessaux Y, Chapelle JP, Vanbelle S, et al. (2007) Long-term prognostic significance of high-sensitivity C-reactive protein before and after coronary angioplasty in patients with stable angina pectoris. Am J Cardiol 99: 31-35.
	67. 	Artieda M, Cenarro A, Ganan A, Lukic A, Moreno E, et al. (2007) Serum chitotriosidase activity, a marker of activated macrophages, predicts new cardiovascular events independently of C-reactive protein. Cardiology 108: 297-306.
	68. 	Imai K, Okura H, Kume T, Yamada R, Miyamoto Y, et al. (2009) C-reactive protein predicts non-target lesion revascularization and cardiac events following percutaneous coronary intervention in patients with angina pectoris. J Cardiol 53: 388-395.
	69. 	Leu HB, Lin CP, Lin WT, Wu TC, Chen JW (2004) Risk stratification and prognostic implication of plasma biomarkers in nondiabetic patients with stable coronary artery disease: the role of high-sensitivity C-reactive protein. Chest 126: 1032-1039.
	70. 	Qi X, Li S, Li J (2003) The prognostic value of IL-8 for cardiac events and restenosis in patients with coronary heart diseases after percutaneous coronary intervention. Jpn Heart J 44: 623-632.
	71. 	Qi X, Li J, Gu J, Li S, Dang Y, et al. (2003) Plasma levels of IL-8 predict early complications in patients with coronary heart disease after percutaneous coronary intervention. Jpn Heart J 44: 451-461.
	72. 	Karha J, Bavry AA, Rajagopal V, Henderson MR, Ellis SG, et al. (2006) Relation of C-reactive protein level and long-term risk of death or myocardial infarction following percutaneous coronary intervention with a sirolimus-eluting stent. Am J Cardiol 98: 616-618.
	73. 	Ikonomidis I, Lekakis J, Revela I, Andreotti F, Nihoyannopoulos P (2005) Increased circulating C-reactive protein and macrophage-colony stimulating factor are complementary predictors of long-term outcome in patients with chronic coronary artery disease. Eur Heart J 26: 1618-1624.
	74. 	Speidl WS, Graf S, Hornykewycz S, Nikfardjam M, Niessner A, et al. (2002) High-sensitivity C-reactive protein in the prediction of coronary events in patients with premature coronary artery disease. Am Heart J 144: 449-455.
	75. 	Haverkate F, Thompson SG, Pyke SDM, Gallimore JR, Pepys MB (1997) Production of C-reactive protein and risk of coronary events in stable and unstable angina. The Lancet 349: 462-466.
	76. 	Thompson SG, Kienast J, Pyke SD, Haverkate F, van de Loo JC (1995) Hemostatic factors and the risk of myocardial infarction or sudden death in patients with angina pectoris. European Concerted Action on Thrombosis and Disabilities Angina Pectoris Study Group. N Engl J Med 332: 635-641.
	77. 	Kubica J, Kozinski M, Krzewina-Kowalska A, Zbikowska-Gotz M, Dymek G, et al. (2005) Combined periprocedural evaluation of CRP and TNF-alpha enhances the prediction of clinical restenosis and major adverse cardiac events in patients undergoing percutaneous coronary interventions. Int J Mol Med 16: 173-180.
	78. 	Kwaijtaal M, van Diest R, Bar FW, van der Ven AJ, Bruggeman CA, et al. (2005) Inflammatory markers predict late cardiac events in patients who are exhausted after percutaneous coronary intervention. Atherosclerosis 182: 341-348.
	79. 	Krzewina-Kowalska A, Kubica J, Kozinski M, Piasecki R, Berent B, et al. (2003) Selected acute phase proteins in patients undergoing coronary angioplasty. Folia Cadiol 10: 733-742.
	80. 	Park DW, Lee CW, Yun SC, Kim YH, Hong MK, et al. (2007) Prognostic impact of preprocedural C reactive protein levels on 6-month angiographic and 1-year clinical outcomes after drug-eluting stent implantation. Heart 93: 1087-1092.
	81. 	Bogaty P, Poirier P, Simard S, Boyer L, Solymoss S, et al. (2001) Biological profiles in subjects with recurrent acute coronary events compared with subjects with long-standing stable angina. Circulation 103: 3062-3068.
	82. 	Zairis MN, Ambrose JA, Manousakis SJ, Stefanidis AS, Papadaki OA, et al. (2002) The impact of plasma levels of C-reactive protein, lipoprotein (a) and homocysteine on the long-term prognosis after successful coronary stenting: The Global Evaluation of New Events and Restenosis After Stent Implantation Study. J Am Coll Cardiol 40: 1375-1382.
	83. 	Aytekin S, Catakoglu AB, Aytekin V, Kocazeybek B, Demiroglu C, et al. (2003) C-reactive protein as a pre-procedural predictor of early and late outcomes of percutaneous coronary interventions. Int Angiol 12: 229-233.
	84. 	Palmerini T, Marzocchi A, Marrozzini C, Ortolani P, Saia F, et al. (2005) Preprocedural levels of C-reactive protein and leukocyte counts predict 9-month mortality after coronary angioplasty for the treatment of unprotected left main coronary artery stenosis. Circulation 112: 2332-2338.
	85. 	Grander W, Dichtl W, Prokop W, Roithinger FX, Moes N, et al. (2004) C-reactive protein plasma levels but not factor VII activity predict clinical outcome in patients undergoing elective coronary intervention. Clin Cardiol 27: 211-216.
	86. 	Marcinkowski M, Czarnecka D, Jastrzebski M, Fedak D, Kawecka-Jaszcz K (2007) Inflammatory markers 10 weeks after myocardial infarction predict future cardiovascular events. Cardiol J 14: 50-58.
	87. 	Kinjo K, Sato H, Sakata Y, Nakatani D, Mizuno H, et al. (2005) Relation of C-reactive protein and one-year survival after acute myocardial infarction with versus without statin therapy. Am J Cardiol 96: 617-621.
	88. 	Kinjo K, Sato H, Ohnishi Y, Hishida E, Nakatani D, et al. (2003) Impact of high-sensitivity C-reactive protein on predicting long-term mortality of acute myocardial infarction. Am J Cardiol 91: 931-935.
	89. 	Lu TM, Ding YA, Lin SJ, Lee WS, Tai HC (2003) Plasma levels of asymmetrical dimethylarginine and adverse cardiovascular events after percutaneous coronary intervention. Eur Heart J 24: 1912-1919.
	90. 	Niccoli G, Ferrante G, Mongiardo R, Perfetti M, Belloni F, et al. (2007) Predictive value of preintervention C-reactive protein on clinical outcome after directional coronary atherectomy followed by stent implantation. Cardiovasc Revasc Med 8: 156-160.
	91. 	Biancari F, Lahtinen J, Lepojarvi S, Rainio P, Salmela E, et al. (2003) Preoperative C-reactive protein and outcome after coronary artery bypass surgery. Ann Thorac Surg 76: 2007-2012.
	92. 	Gaspardone A, Crea F, Versaci F, Tomai F, Pellegrino A, et al. (1998) Predictive value of C-reactive protein after successful coronary-artery stenting in patients with stable angina. Am J Cardiol 82: 515-518.
	93. 	Patti G, Di SG, D'Ambrosio A, Dicuonzo G, Abbate A, et al. (2002) Prognostic value of interleukin-1 receptor antagonist in patients undergoing percutaneous coronary intervention. Am J Cardiol 89: 372-376.
	94. 	Huang W, Lei H, Liu Q, Ma KH, Qin S, et al. (2009) Combination of C-reactive protein and cardiac troponin I for predicting adverse cardiac events after sirolimus-eluting stent implantation. Coron Artery Dis 20: 245-250.
	95. 	Palmerini T, Marzocchi A, Marrozzini C, Reggiani LB, Savini C, et al. (2007) Preoperative C-reactive protein levels predict 9-month mortality after coronary artery bypass grafting surgery for the treatment of left main coronary artery stenosis. Eur J Cardiothorac Surg 31: 685-690.
	96. 	Soeki T, Tamura Y, Shinohara H, Tanaka H, Bando K, et al. (1999) Fibrinolytic factors, serum lipid and C-reactive protein predicting cardiac events in Japanese patients with coronary atherosclerotic lesions. Jpn Circ J 63: 976-980.
	97. 	van der Harst P, Voors AA, Volbeda M, Buikema H, van Veldhuisen DJ, et al. (2006) Usefulness of preoperative C-reactive protein and soluble intercellular adhesion molecule-1 level for predicting future cardiovascular events after coronary artery bypass grafting. Am J Cardiol 97: 1697-1701.
	98. 	Huang W, Chen QW, Lei H, Deng W, Ke DZ (2006) Predictive value of fibrinogen and high-sensitivity C-reaction protein for cardiovascular events in patients with stable coronary artery disease. Zhonghua Xin Xue Guan Bing Za Zhi 34: 718-721.
	99. 	Milazzo D, Biasucci LM, Luciani N, Martinelli L, Canosa C, et al. (1999) Elevated levels of C-reactive protein before coronary artery bypass grafting predict recurrence of ischemic events. Am J Cardiol 84: 459-61, A9.
	100. 	Morrow DA, de Lemos JA, Sabatine MS, Wiviott SD, Blazing MA, et al. (2006) Clinical relevance of C-reactive protein during follow-up of patients with acute coronary syndromes in the Aggrastat-to-Zocor Trial. Circulation 114: 281-288.
	101. 	Haim M, Benderly M, Tanne D, Matas Z, Boyko V, et al. (2007) C-reactive protein, bezafibrate, and recurrent coronary events in patients with chronic coronary heart disease. Am Heart J 154: 1095-1101.
	102. 	Palazzuoli A (2006) Brain Natriuretic Peptide and Other Risk Markers for Outcome Assessment in Patients With Non-ST-Elevation Coronary Syndromes and Preserved Systolic Function. Am J Cardiol 
	103. 	Schaan BD, Quadros AS, Sarmento-Leite R, De LG, Jr., Bender A, et al. (2007) 'Correction:' Serum transforming growth factor beta-1 (TGF-beta-1) levels in diabetic patients are not associated with pre-existent coronary artery disease. Cardiovasc Diabetol 6: 19.
	104. 	Chirinos JA, Zambrano JP, Chakko S, Schob A, Veerani A, et al. (2005) Usefulness of C-reactive protein as an independent predictor of death in patients with ischemic cardiomyopathy. Am J Cardiol 95: 88-90.
