# Supplementary material for: Evaluating the Quality of Research into a Single Prognostic Biomarker: A Systematic Review and Meta-analysis of 83 Studies of C-Reactive Protein in Stable Coronary Artery Disease
Source: PLoS Med. 2010 Jun 1;7(6):e1000286. doi: 10.1371/journal.pmed.1000286 (PMC2879408; doi:10.1371/journal.pmed.1000286)
Supplement: Table S2 — Definitions of 17 items of study reporting quality. (0.06 MB RTF) [file pmed.1000286.s003.rtf]

Table S2. Definitions of 17 items of study reporting quality 
REMARK Guidelines 2006[12]	Label for item	How the item was operationalised	
“Give rationale for sample size”	Rationale for sample size 
	Was there a statistical sample size or power calculation 	
“State any pre-specified hypotheses”	Pre-specified hypothesis  or study protocol	Was there a bibliographic reference (e.g. to a protocol) stating that studying the relation of circulating biomarkers with coronary events was part of the rationale for collecting the patient sample; or was there a pre-specified analysis plan?	
Population			
“Describe source of patients”	Healthcare setting	What was the healthcare setting from which patients were recruited? 	
“Describe inclusion and exclusion criteria”	Exclusion criteria	Were exclusion criteria reported?

	
“Describe number of patients included in each stage of the analysis and reasons for dropout”	Number of patients included in each stage of the analysis and reasons for dropout	Was there a description of numbers of patients at different stages (a. the total number of patients who were invited to participate in the study and who met eligibility criteria, b. The subset with complete follow up and the reasons for dropout.
	
“Describe disease stage”	Time between initial presentation of coronary disease and CRP measurement  	Was the average length of time between the first symptomatic clinical presentation of coronary disease and CRP measurement stated? OR Was the minimum length of time since diagnosis with coronary disease stated?	
Biomarker measurement			
“Specify assay method and provide (or reference) protocol	Manufacturer	Was the name of the company which makes the assay for CRP stated?  	
	Assay 	Was the type of assay used to measure CRP described?	
“Describe methods of storage	Sample handling	Was CRP measured in a fresh sample or if the blood was stored, was the temperature stated?	
Confounders			
“Report standard prognostic variables ”	Conventional risk factors and inflammatory markers  measured	Were the following conventional risk factors  measured:  age, sex, smoking status, total cholesterol, low-density lipoprotein cholesterol, high density lipoprotein cholesterol, triglycerides, body mass index, diabetes AND was at least one inflammatory marker measured (e.g. fibrinogen, IL-6 or white cell count)	
Outcomes			
“Precisely define all clinical endpoints”	Primary outcome 	Was a single disease outcome, or a single combination of outcomes, defined as the primary outcome for the analysis?	
	Validation	Were outcome events cross checked by independent sources? E.g. examining clinical records and national routine data	
“Specify whether, and how, assays were performed blinded to the study endpoint”	Masking	Was the ascertainment and classification of outcomes blinded to the CRP value and other clinical information?	
Analytic decisions			
“Present univariate analyses of relation between marker and outcome”	Univariate estimate	Was the effect of CRP on outcome presented either crude, or adjusted for age, or adjusted for age and sex?	
“Provide estimated effects... in which the marker and standard prognostic variables are included, regardless of their statistical significance”
“Report distributions”	Adjusted for all conventional risk factors	Were the conventional risk factors (listed above) included as adjustments, regardless of their statistical significance? 	
“Clarify how marker values were handled in the analysis”	Comparison group rationale	Was a reason given for choosing to analyse the data as continuous or categorical and if cutpoints were used was the method for their selection clear? 	
“How were missing data handled”	Missing values	a. Was the number of patients with missing values for CRP or confounders stated?  AND 
b. Was it stated how missing values were dealt with in the analysis? 	
