# Supplementary material for: Evaluating the Quality of Research into a Single Prognostic Biomarker: A Systematic Review and Meta-analysis of 83 Studies of C-Reactive Protein in Stable Coronary Artery Disease
Source: PLoS Med. 2010 Jun 1;7(6):e1000286. doi: 10.1371/journal.pmed.1000286 (PMC2879408; doi:10.1371/journal.pmed.1000286)
Supplement: Table S3 — Adjusting for publication bias using different methods as previously described [25]. (0.06 MB RTF) [file pmed.1000286.s004.rtf]

Table S3. Adjusting for publication bias using different methods as previously described[1]

	Meta-Analysis
Model1	Regression
Covariate2	Weight3	Include a Dispersion 
Parameter	RR (Confidence interval)	
MA (No adjustment)	

FE	
None	
1/Var	
No	1.25 (1.21-1.29)	
MA (No adjustment)	

RE	
None	
1/Var	
No	1.97 (1.78-2.17)	
Trim & Fill 	

'Trim' FE	
None	
1/Var	
No	1.20 (1.16-1.23)	
Trim & Fill 	
'Fill' FE
'Trim' FE	

None	

1/Var	

No	1.37 (1.24-1.52)	
Trim & Fill 	
'Fill' RE
'Trim' RE	

None	

1/Var	

No	1.63 (1.47-1.79)	
Egger Dispersion (original)	
'Fill' RE
FE	

SE	

1/Var	

Yes	1.03 (0.98-1.08)	
Egger FE 	

FE	
SE	
1/Var	
No	1.03 (0.99-1.07)	
Egger RE 	

RE	
SE	
1/Var	
No	1.08 (0.97-1.20)	
EggerVar Dispersion 	

FE	
Var	
1/Var	
Yes	1.19 (1.13-1.25)	
EggerVar FE 	

FE	
Var	
1/Var	
No	1.19 (1.15-1.22)	
EggerVar RE 	

RE	
Var	
1/Var	
No	1.56 (1.41-1.72)	
Peters 	

FE	
1/SS	
1/VarP	
Yes	1.59 (1.39-1.82)	
Macaskill  	

FE	
1/SS	
1/VarP	
Yes	1.18 (1.08-1.28)	
Conditional EggerVar Disp	

FE	
Var	
1/Var	
Yes	1.19 (1.13-1.25)	
						

Abbreviations: RR, relative risk; 1 Meta-analysis model: FE, fixed effects; MA, meta-analysis; RE, random effects; 2 Regression covariate: SE Standard Error, Var within-study variance, SS Sample Size; 3 Weight: Var observed within-study variance or sum of within-study variance and between-study variance in RE meta-analysis model, VarP variance of the proportion of the number of events in a study.
MA (No adjustment) RE model is the random effects meta-analysis summary estimate used in this paper, without adjustment for publication bias.

References

1. 	Moreno SG, Sutton AJ, Ades AE, Stanley TD, Abrams KR, et al. (2009) Assessment of regression-based methods to adjust for publication bias through a comprehensive simulation study. BMC Med Res Methodol 9: 2.
