# Supplementary material for: Evaluating the Quality of Research into a Single Prognostic Biomarker: A Systematic Review and Meta-analysis of 83 Studies of C-Reactive Protein in Stable Coronary Artery Disease
Source: PLoS Med. 2010 Jun 1;7(6):e1000286. doi: 10.1371/journal.pmed.1000286 (PMC2879408; doi:10.1371/journal.pmed.1000286)
Supplement: Text S1 — Checklist summarising compliance with MOOSE guidelines. (0.06 MB DOC) [file pmed.1000286.s005.doc]

CRP Meta-analysis: Checklist summarising compliance with MOOSE guidelines

| **Reporting background should include** | |
| --- | --- |
| Problem definition | Yes |
| Hypothesis statement | Yes |
| Description | Yes |
| Type of exposure or intervention used | Yes |
| Type of study designs used | Yes |
| Study population | Yes |
| **Reporting of search strategy should include** | |
| Qualifications of searches (e.g. librarians and investigators) | Yes |
| Search strategy, including time period included in the synthesis and keywords | Yes |
| Effort to include all available studies, including contact with authors | Yes |
| Databases and registries searched | Yes |
| Search software used, name and version, including special features | Yes |
| Use of hand searching (e.g. reference lists of obtained articles) | No |
| List of citations located and those excluded including justification | Available on request |
| Method of addressing articles published in languages other than English | Yes |
| Method of handling abstracts and unpublished studies | No |
| Description of any contact with authors | No |
| **Reporting methods should include** | |
| Description of relevance or appropriateness of studies assembled for assessing the hypothesis to be tested | Yes |
| Rationale for the selection and coding of data (eg, sound clinical principles or convenience) | Yes |
| Documentation of how data were classified and coded (eg, multiple raters, blinding, and interrater reliability) | Yes |
| Assessment of confounding (eg, comparability of cases and controls in studies where appropriate) | Yes |
| Assessment of study quality, including blinding of quality assessors; stratification or regression on possible predictors of study results | Yes |
| Assessment of heterogeneity | Yes |
| Description of statistical methods (eg, complete description of fixed or random effects models, justification of whether the chosen models account for predictors of study results, dose-response models, or cumulative meta-analysis) in sufficient detail to be replicated | Yes |
| Provision of appropriate tables and graphics | Yes |
| **Reporting of results should include** | |
| Graphic summarizing individual study estimates and overall estimate | Yes |
| Table giving descriptive information for each study included | Yes |
| Results of sensitivity testing (eg, subgroup analysis) | Yes |
| Indication of statistical uncertainty of findings | Yes |
| **Reporting of discussion should include** | |
| Quantitative assessment of bias (eg, publication bias) | Yes |
| Justification for exclusion (eg, exclusion of non–English-language citations) | Yes |
| Assessment of quality of included studies | Yes |
| **Reporting of conclusions should include** | |
| Consideration of alternative explanations for observed results | Yes |
| Generalization of the conclusions (ie, appropriate for the data presented and within the domain of the literature review) | Yes |
| Guidelines for future research | Yes |
| Disclosure of funding source | Yes |
